# Co-ordinated activation of classical and novel PKC isoforms is required for PMA-induced mTORC1 activation

**DOI:** 10.1371/journal.pone.0184818

**Published:** 2017-09-19

**Authors:** Mengling Liu, Christopher J. Clarke, Mohamed F. Salama, Yeon Ja Choi, Lina M. Obeid, Yusuf A. Hannun

**Affiliations:** 1 Department of Medicine, Stony Brook University, Stony Brook, NY, United States of America; 2 Stony Brook Cancer Center, Stony Brook University Hospital, Stony Brook, NY, United States of America; 3 Department of Biochemistry, Faculty of Veterinary Medicine, Mansoura University, Mansoura, Egypt; 4 Department of Pathology, Stony Brook University, Stony Brook, NY, United States of America; Emory University, UNITED STATES

## Abstract

Protein kinase C (PKC) has been shown to activate the mammalian target of rapamycin complex 1 (mTORC1) signaling pathway, a central hub in the regulation of cell metabolism, growth and proliferation. However, the mechanisms by which PKCs activate mTORC1 are still ambiguous. Our previous study revealed that activation of classical PKCs (cPKC) results in the perinuclear accumulation of cPKC and phospholipase D2 (PLD2) in recycling endosomes in a PLD2-dependent manner. Here, we report that mTORC1 activation by phorbol 12,13-myristate acetate (PMA) requires both classic, cPKC, and novel PKC (nPKC) isoforms, specifically PKCη, acting through distinct pathways. The translocation of mTOR to perinuclear lysosomes was detected after treatment of PKC activators, which was not colocalized with PKCα- or RAB11-positive endosomes and was not inhibited by PLD inhibitors. We found that PKCη inhibition by siRNA or bisindolylmaleimide I effectively decreased mTOR accumulation in lysosomes and its activity. Also, we identified that PKCη plays a role upstream of the v-ATPase/Ragulator/Rag pathway in response to PMA. These data provides a spatial aspect to the regulation of mTORC1 by sustained activation of PKC, requiring co-ordinated activation of two distinct elements, the perinuclear accumulation of cPKC- and PLD-containing endosomes and the nPKC-dependent translation of of mTOR in the perinuclear lysosomes. The close proximity of these two distinct compartments shown in this study suggests the possibility that transcompartment signaling may be a factor in the regulation of mTORC1 activity and also underscores the importance of PKCη as a potential therapeutic target of mTORC-related disorders.

## Introduction

The mammalian target of rapamycin (mTOR) is a crucial signaling hub in eukaryotes, functioning to sense and integrate environmental changes such as alterations in nutrients, growth factors, energy stress and oxygen levels into cellular responses [1]. Currently, mTOR is known to exist in two distinct complexes, mTOR complex 1 (mTORC1) and mTOR complex 2 (mTORC2) through interaction with different protein partners. Of these, mTORC1 is the most studied and regulates translation, proliferation, cell size and autophagy through its downstream effectors including ribosomal S6 kinase (S6K), 4E-BP1 and ULK [2].

Given this central position, the regulation of mTORC1 is understandably complex and involves a variety of factors depending on the stimulus. Thus, amino acids regulate mTORC1 through the RAG GTPases [3] that recruit mTOR to the lysosomal surface and in proximity to its activator, the small G-protein Rheb [4, 5] with subsequent studies identifying the Ragulator complex [4] GATOR complexes, vacuolar H^+^-ATPase (v-ATPase) [6], folliculin, and sestrins as upstream regulators of the Rag pathway [4, 6–8]. More recently, studies have also identified amino acid regulation of mTORC1 through Rag-independent pathways involving the class III phosphinositide 3-kinase Vps34 and phospholipase D (PLD) [9]. By contrast, growth factors and glucose promote mTORC1 primarily through the Rheb pathway. In this pathway, the tuberin-hamartin (TSC1/2) complex functions as a GAP for Rheb, and considerable research has shown that multiple signals converge on TSC1/2 to suppress or promote mTORC1 activity [10]. For example, phosphorylation by AMP kinase (in the glucose pathway) or glycogen synthase kinase-3B (GSK3B) (in the Wnt pathway) activates TSC1/2 and inhibits mTORC1 [11, 12]. In contrast, phosphorylation by Akt (in the growth factor pathway) inhibits TSC1/2, thereby activating mTORC1 [13]. More recently, translocation of TSC1/2 on and off the lysosomal surface (where it colocalizes with RHEB) was identified as a primary regulatory mechanism in response to growth factors [14], thus defining a spatial aspect to regulation through the TSC1/2-Rheb arm of the pathway.

Protein kinase C (PKC) is a family of 10 isoforms grouped into 3 subfamilies (classical, novel, and atypical) based on their structures and activators [15]. Classical isoenzymes of PKCs (cPKCs: α, βI, βII and γ), are activated by diacylglycerol (DAG) and calcium whereas novel PKCs (nPKCs: δ, ε, η and θ) are DAG-dependent but calcium-independent [15]. In contrast, the atypical PKCs (ζ and ι/λ) are independent of both DAG and calcium. Additionally, both cPKC and nPKC isoforms are activated by tumor promoting phorbol esters such as phorbol-12-myristate-13-acetate (PMA)–which function by mimicking DAG [16]. Acute activation of cPKCs occurs in response to stimulation of phospholipase C by growth factor receptors or G-protein coupled receptors (GPCRs) and generation of DAG, resulting in the rapid translocation of cPKCs from the cytosol to the plasma membrane [17]. This allows PKC to phosphorylate local substrates and activate downstream signaling but is relatively short lived and, following metabolism of DAG, PKC returns to the cytosol in a mechanism requiring autophosphorylation [18, 19].

In contrast to this well-established paradigm, we have previously reported that sustained activation of cPKCs by PMA or GPCRs results in internalization and translocation of cPKCs to a perinuclear subset of RAB11-positive recycling endosomes (which we termed the ‘pericentrion’) [20–22]. Notably, the perinuclear accumulation of PKC required sustained activities of cPKCs and PLD and was dependent on caveolae- and clathrin-mediated endocytosis [23]. Functionally, in addition to PKC itself, the pericentrion also contained PLD, lipids and some receptors (e.g. serotonin receptor, epidermal growth factor receptor) and was important for heterologous desensitization of growth factor receptors [20]. More intriguingly, we found that the majority of PKC-dependent phosphorylation in response to sustained PKC activation was temporally concurrent with perinuclear PKC accumulation and required endocytosis, PKC and PLD activity. Importantly, one such downstream effector of PKC was S6K– the best characterized substrate of mTORC1 [24].

Considerable evidence has implicated the mTOR pathway as a downstream effector of PKC signaling in various cell lines. For example, in adult cardiac muscle cells, PKCε and PKCδ are required for ET-1 induced mTORC1 activation, and PKCδ is involved in insulin-stimulated activation of mTORC1 [25]. In glioma cells, increases in EGFR protein levels correlated with elevated activity of PKC and mTORC1, and signals from EGFR to mTORC1 were through PKCα and independent of AKT [26], while in glioblastoma cells, PKCη was involved in mTOR activation and proliferation [27]. Our own recent study found that high PKCβII levels were associated with increased mTORC1 activity in lung cancer cells and overexpression of PKCβII in HEK293 cells was sufficient to activate mTORC1. Notably, this increased mTORC1 activity and subsequent increased proliferation and invasion were all PLD-dependent [28]. Finally, PKCξ, an atypical PKC, was implicated in abnormal mTOR regulation in follicular lymphoma cells in a MAPK-dependent manner [29]. In addition to these, numerous studies have shown that PMA activates mTORC1 in a PKC-dependent manner and have further explored the underlying mechanisms. To date, this has heavily focused on the MAPK pathway and it was reported that PMA activates mTOR through ERK regulation of 90-kDa ribosomal S6 kinases (RSK) [30]. However, a more recent study found that RSK was dispensable for PMA-induced mTORC1 activation, and the requirement of MEK/ERK in this process is variable across cell lines [31] suggesting alternative mechanisms by which PMA and, by extension, PKC regulates mTORC1.

In this study, we have investigated the mechanisms by which sustained PMA treatment regulates the activation of mTORC1. We find that PMA activation of mTORC1 involves dual pathways and mechanisms. The first pathway requires the formation of the cPKC-containing endosomes through activation of PLD as we have described previously. The second, previously unknown pathway requires translocation of mTORC1 to the lysosomal surface through activation of the novel PKCη. These data identified a hitherto unknown regulator of mTORC1. Given the tendency of this pathway to be hyperactivated in many cancers, the possibility of targeting PKCη as a means to disrupt this activity warrants further investigation.

## Materials and methods

### Materials

All cell lines were purchased from American Tissue Culture Collection (Manassas, VA, USA). Lipofectamine RNAiMAX were from Life Technologies (Grand Island, NY). X-treme GENE 9 were from Roche (Indianapolis, IN). PLD2 inhibitor, VU 0364739 was a kind gift from Dr. Alex Brown (Vanderbilt University, Nashville, TN, USA). PMA, Gö 6976, Bisindolylmaleimide I and U0126 were from EMD Millipore (Billerica, MA). Anti-phospho-p70S6K (Thr389), S6K, mTOR, phospho-ERK1/2 (Thr202/Tyr204), ERK1/2, EGFR, HA antibodies were from Cell Signaling Technology (Danvers, MA) and anti-Actin antibody was purchased from Sigma Aldrich (St. Louis, MO). Anti-LAMP1, LAMP2, PKCδ and PKCε antibodies were from Santa Cruz Biotechnology (Santa Cruz, CA). Anti-EEA1 antibody was from BD Biosciences (San Jose, CA). siRNA for PKCη, PKCδ, PKCε, RAGB, LAMTOR 1, and LAMTOR 3 were from Qiagen (Valencia, CA).

### Cell culture

HEK293 cells were maintained in MEM supplemented with 10% (V/V) fetal bovine serum. HeLa cells were grown in DMEM supplemented with 10% (V/V) fetal bovine serum. All cells were grown at 37°C in a humidified atmosphere with 5% CO2.

### Plasmid construction and siRNA transfection

The wild type pBK-CMV-GFP-PKCα was described previously [32] and was transfected into cells using Xtremegene 9 (Roche) according to the manufacturers protocol. siRNAs were transfected into HEK293 or HeLa cells with Lipofectamine RNAiMAX following the manufacturers protocol. Transfected cells were grown in the growth medium for 72 hours prior to treatment.

### Immunoblotting

Protein samples were collected in LDS sample buffer (Invitrogen) and boiled for 10 min. Proteins were separated on 4–20% polyacrylamide gels (BioRad), transferred to nitrocellulose membranes (BioRad), which were blocked in PBS-T with 5% non-fat dried milk for 1 hour at room temperature. After three washes with PBS-T, membranes incubated with primary antibody overnight at 4°C. Primary antibody solutions were prepared according to manufacturer’s recommendations. On the following day, blots were washed three times (PBS-T) and incubated with HRP-conjugated secondary antibodies in blocking solution for 1 hour. After three washes, proteins were detected with enhanced chemiluminescence reagent (Pierce).

### Indirect immunofluorescence and confocal microscopy

Cells were plated on 35-mm confocal dishes (MatTek) at a density 3 to 5 ×10^5^ cells/dish. After 24 hours, cells were transfected as described above. After transfection, cells were grown in for 24 to 48 hours before serum starvation for 5 hours prior to treatment. Cells transfected with green fluorescence protein (GFP) were fixed with 3.7% paraformaldehyde and nuclei were stained with DAPI. Indirect immunofluorescence was performed as described before [20]. All images were taken by Leica TCS SP8, and pictures are representative of at least five fields taken from three independent experiments. To quantify colocalization, the images were analyzed using Image J coloc2.

### Proximity ligation assay

HeLa cells were plated on 35-mm confocal dishes at a density 3–5 ×10^5^ cells/dish. After 24 to 48 hours, cells were starved for 5 hours followed by treatment with vehicle or PMA as shown. Cells were then fixed and permeabilized as described before. After incubation with primary antibodies (LAMP1 and mTOR), the proximity ligation assay (PLA) was performed as described previously [28]. The signal from each detected pair of PLA probes was acquired with a Leica TCS SP8 microscope and the quantifications were performed from at least 3 images. PLA signals were counted by Image J and normalized by the cell number.

### Statistical analysis

Statistical significance was calculated with student’s t-test or by two-way ANOVA with Bonferroni Post test where appropriate. A p-level below 0.05 was considered to be statistically significant.

## Results

### PMA induces mTOR accumulation in perinuclear lysosomes

**Fig 1:**
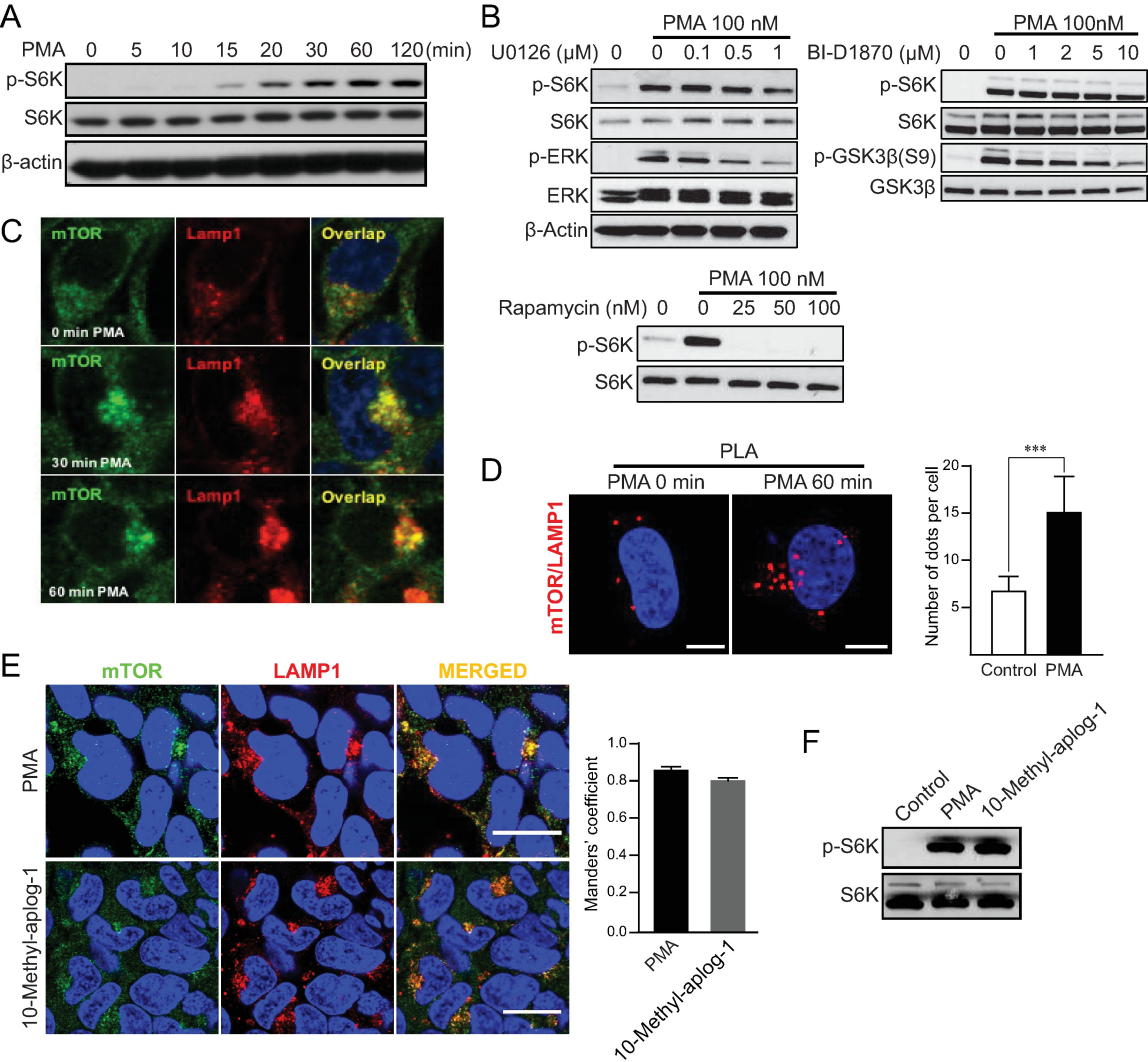
PMA activates mTOR and induces its accumulation in perinuclear lysosomes. (A) Time dependent changes of phosphorylated and total S6K in HEK293 cells treated with 100 nM PMA 5 hours after serum starvation. (B) The levels of S6K, ERK, and GSK3β were evaluated in HEK293 cells pretreated with MEK inhibitor (U0126), RSK inhibitor (BI-D1870) or rapamycin for 1 hour at the indicated concentrations, prior to treatment with 100 nM PMA for 1 hour. (C) Confocal microscope images of immunofluorescence labeling of mTOR and LAMP1 in HEK293 cells treated with 100 nM PMA for 30 or 60 min 5 hours after serum starvation. Scale bar, 10 μm (D) PLA to evaluate the proximity of mTOR and LAMP1 in HeLa cells treated with 100 nM PMA 5 hours after serum starvation. Bar graph in (D) represents the number of dots per cell obtained from three independent experiments. *** *P* < 0.001; Student T-Test). Scale bar, 10 μm. (E) Confocal microscope images of immunofluorescence labeling of mTOR and LAMP1, and (F) the levels of phosphorylated and total S6K in HEK293 cells treated with 100 nM PMA or 500 nM 10-methyl-aplog-1 for 1 hour following serum starvation for 5 hours. Scale bar in (E), 20 μm. Images are representative of three fields examined from three independent experiments. Western blots are representative of at least three independent experiments.

Considerable research has implicated mTORC1 as a downstream effector of PKC signaling [25–29]. Previously, we reported that sustained activation of cPKCs by PMA induced mTORC1 activation–as assessed by S6K phosphorylation–with delayed kinetics, requiring endocytosis and PLD2 activity [24, 28]. Here, we have explored the underlying mechanisms of this regulatory axis further. To confirm the kinetics of S6K phosphorylation by sustained activation of cPKCs, a detailed time course of PMA treatment was performed. HEK293 cells were treated with PMA for 5, 10, 15, 20, 30, 60 and 120 min, and phosphorylation status of S6K at Thr389 was analyzed (**Fig 1A**). As can be seen, the phosphorylation of S6K started 15 min and achieved a maximum at 60 min after PMA treatment. To confirm that the observed increase in S6K phosphorylation was mediated by mTORC1, cells were pre-treated with rapamycin prior to PMA stimulation (**Fig 1B**). As can be seen, there was complete inhibition of PMA-induced S6K phosphorylation by rapamycin confirming this phosphorylation was mediated by mTORC1.

While early research implicated the MEK/ERK pathway in PMA activation of mTORC1 through activation of RSK [30], a more recent study reported that the role of MEK/ERK for mTORC1 activation is variable among different cell lines and that RSK was not required for PMA-induced mTORC1 activation [31]. To clarify the role of MEK/ERK and RSK in our experimental system, cells were pre-treated with the MEK inhibitor U0126 or the RSK inhibitor BI-D1870 prior to PMA treatment. Results showed that while 1 μM U0126 was able to inhibit PMA-induced ERK phosphorylation by more than 70%, this concentration had minor effects on S6K phosphorylation (**Fig 1B**). Similarly, 10µM BI-D1870 decreased PMA-induced GSK3β phosphorylation by more than 75% with no effect on S6K phosphorylation (**Fig 1B**). Taken together, these results confirmed that MEK/ERK and RSK are largely dispensable for activation of mTORC1 induced by PMA in our system.

Accumulating evidence has suggested that the lysosomal localization of mTORC1 is essential for its full activation by bringing mTORC1 in close proximity to its upstream activator Rheb [3]. Despite this, the effects of PMA on mTORC1 translocation have not been determined. Analysis of mTOR and LAMP1 by confocal microscopy found some basal colocalization was present, consistent with previous studies [33]. More strikingly, following prolonged PMA treatment, mTOR clustered in the perinuclear region where it strongly colocalized with LAMP1 (**Fig 1C**). Moreover, the degree of colocalization of mTOR and LAMP1 increased with longer PMA treatment (**Fig 1C**). To consolidate these results in a more quantifiable manner, PLA were performed to evaluate the interaction of mTOR with LAMP1. PMA significantly increased the proximity of mTOR and Lamp1 compared to the vehicle treatment (**Fig 1D**) suggesting that PMA increased localization of mTORC1 to the lysosomal surface.

In addition, we confirmed these results using another PKC activator, 10-methyl-aplog-1 [34] to prove the activation and translocation of mTOR are dependent on PKC, not specific to PMA. 10-Methyl-aplog-1 also induced both translocation of mTOR to perinuclear lysosomes and S6K phosphorylation to the same extent as PMA (**Fig 1E and 1F**). Taken together, these results suggested that the sustained activation of PKCs induces mTORC1 translocation to lysosomes.

### Lysosomal mTOR is adjacent to, but distinct from, the perinuclear cPKC-containing endosomes

**Fig 2:**
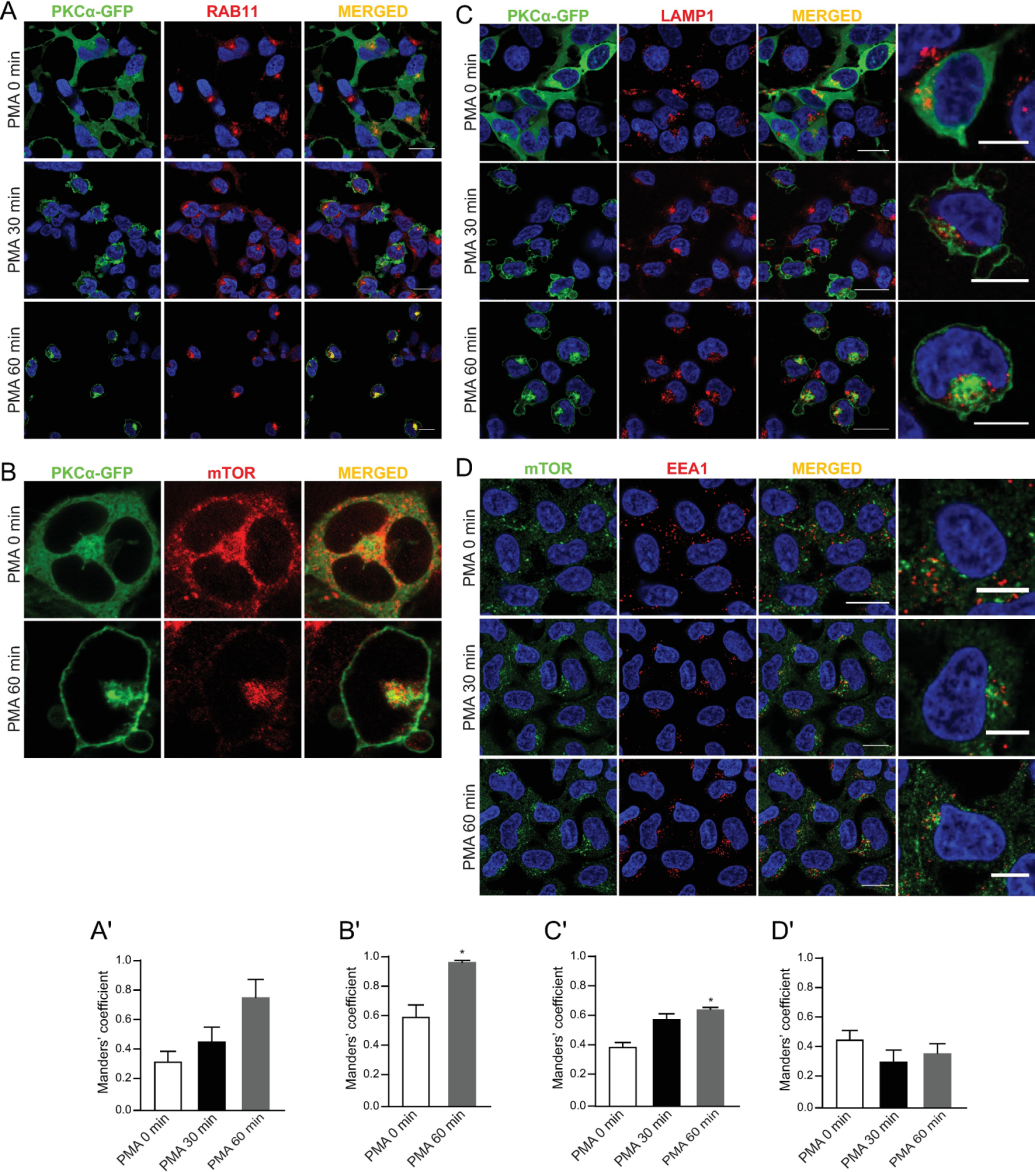
Lysosomal mTOR is adjacent to, but distinct from, the perinuclear cPKC-containing endosomes. Confocal microscopy images of immunofluorescence labeling of (A) GFP and RAB11, (B) GFP and mTOR, (C) GFP and LAMP1, and (D) mTOR and EEA1 in HEK293 cells were transfected with PKCα-GFP and starved for 5 hours prior to treatment with 100 nM PMA for 30 or 60 min. Images are representative of three fields examined from three independent experiments. Scale bar, 20 μm in (A-B), 10 μm in enlarged images in (C and D).

We have reported that sustained stimulation of PKC with PMA leads to sequestration of recycling endosomes and cPKCs into a RAB11-positive juxtanuclear compartment [21]. We speculated that cPKC and mTORC1 may colocalize in the perinuclear region following PMA treatment, and this may be functionally important for mTORC1 activation by PMA. Consistent with our previous observations, PMA treatment induced translocation of PKCα-GFP from the cytoplasm to the perinuclear region where it co-localized with RAB11-positive endosomes (**Fig 2A and 2A’**). Strikingly, upon PMA treatment, both PKCα-GFP and mTOR localized to the perinuclear region (**Fig 2B**) with some overlap between the two proteins indicated (**Fig 2B’**). Similar overlap was observed with PKCα-GFP and LAMP1 upon PMA treatment (**Fig 2C and 2C’**). This raised the possiblity that, while the mTOR-containing lysosomes and cPKC-containing endosomes are both in the perinuclear region, they are distinct compartments. To confirm this, we examined the localization of mTOR and EEA1 at earlier time points as we had previously observed the trafficking of PKCα-GFP through the early endosomes en route to the perinuclear region [20, 35]. As we anticipated, mTOR did not colocalize with EEA1 at any of the time points investigated (**Fig 2D and 2D’**), further supporting the hypothesis that mTOR and PKCα are present in distinct compartments.

**Fig 3:**
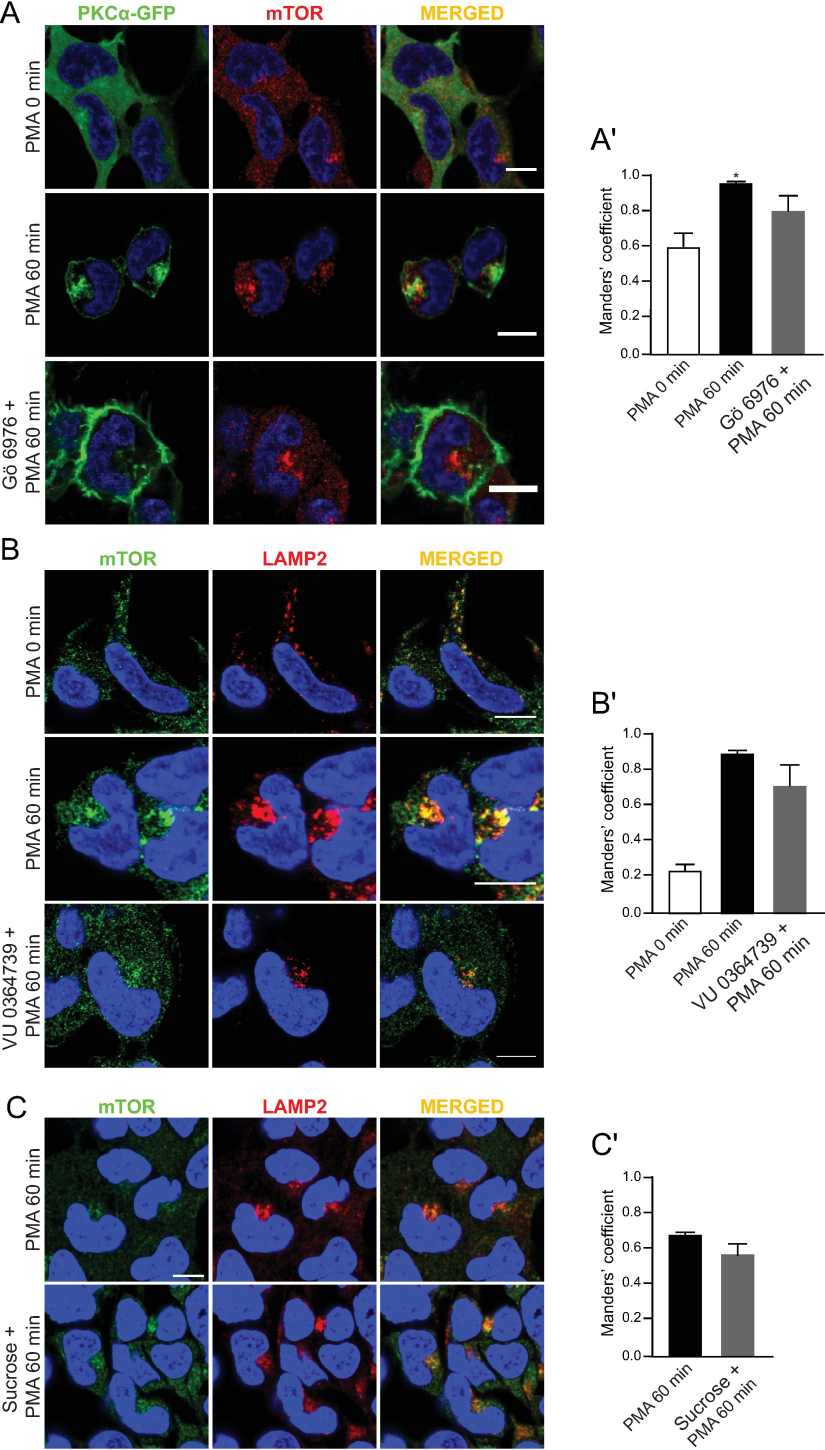
Formation of the perinuclear cPKC-containing endosomes, but not perinuclear mTOR-containing lysosomes, requires endocytosis, cPKC, and PLD activity. (A) Confocal microscopy images of immunofluorescence labeling of GFP and mTOR in PKCα-GFP-overexpressing HEK293 cells pretreated with 3uM Gö 6976 for 1 hour followed by treatment with 100nM PMA for 1 hour. Confocal microscope images of immunofluorescence labeling of mTOR and lysosomal markers (LAMP1 or LAMP2) in HEK293 cells pretreated with (B) 3 uM PLD2 inhibitor, VU 0364739 for 1 hour or (C) 400 mM sucrose for 30 min followed by treatment with 100nM PMA for 1 hour. Images are representative of three fields examined from three independent experiments. Scale bar, 10 μm in (A-C). (A’-C’) Quantification of co-localization for the respective experiments.

To consolidate these data, we took advantage of our previous observations that the formation of the perinuclear cPKC-containing endosomes requires endocytosis, as well as PKC and PLD activity. Accordingly, cells were pre-treated with a cPKC inhibitor (Gö 6976), PLD inhibitor (1-butanol), PLD2 inhibitor (VU 0364739), or endocytosis inhibitor (hypertonic sucrose) prior to stimulation with PMA. Notably, none of these inhibitors affected PMA-induced perinuclear translocation of mTOR and its colocalization with lysosome markers (**Fig 3A–3C and 3A’–3C’**), despite preventing perinuclear translocation of PKCα-GFP (**Fig 3A**). Importantly, we have previously shown that these inhibitors blocked PMA-induced S6K phosphorylation [24, 28]. Collectively, these results indicated that upon PMA stimulation, mTOR accumulates in the perinuclear lysosomes coincident with the formation of cPKC-containing endosomes. These data suggested that perinuclear localization of cPKC-containing endosomes is important for full activation of mTORC1 in response to sustained PKC activation.

### Novel PKCeta is required for mTORC1 activation through regulating mTOR localization

**Fig 4:**
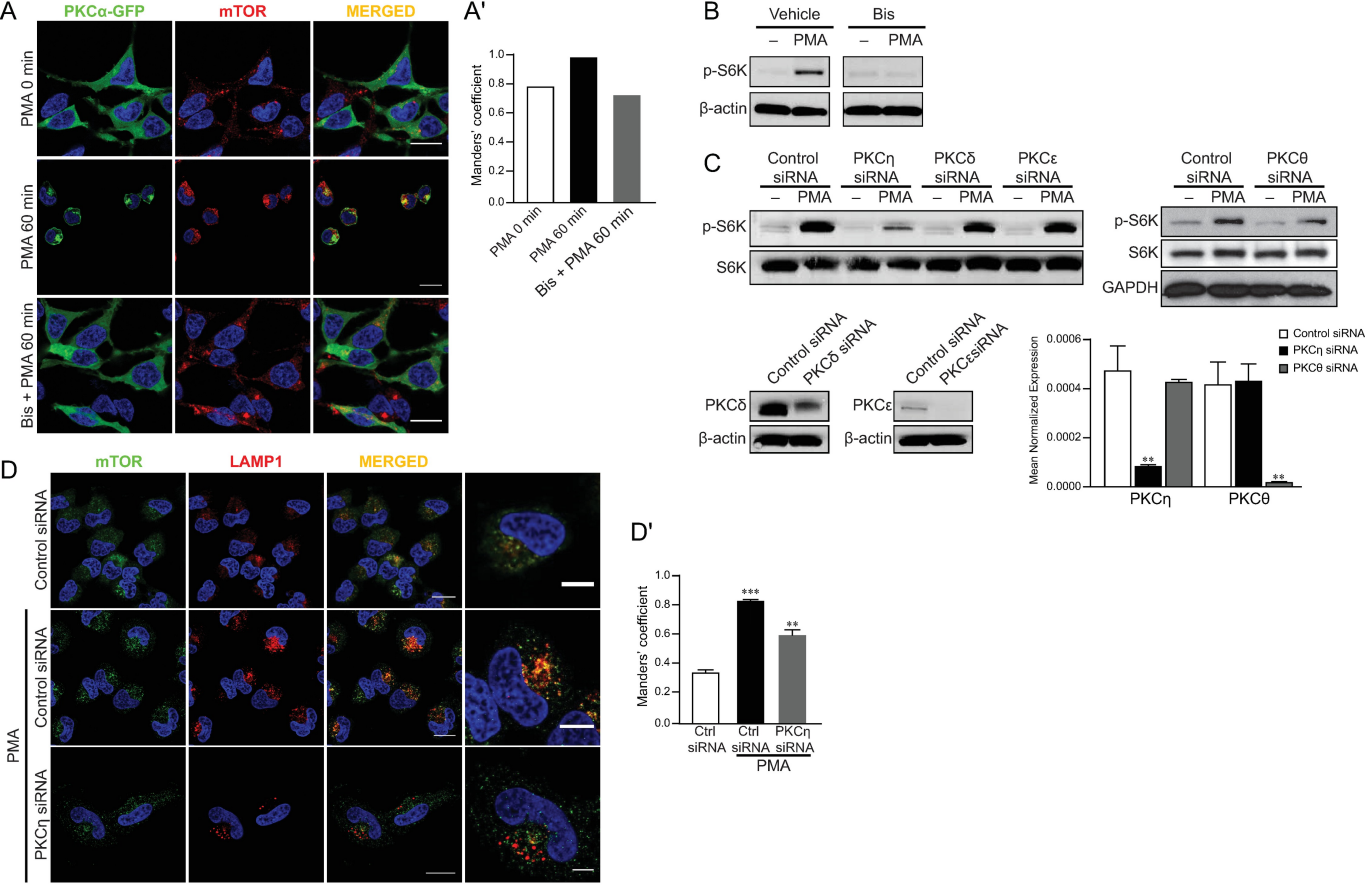
PKCη is required for mTORC1 activation by regulating mTOR localization. (A) Confocal microscopy images of immunofluorescence labeling of GFP and mTOR in PKCα-GFP-overexpressed HEK293 starved for 5 hours and then pretreated with 3 uM Bis for 1 hour followed by 100 nM PMA for 1 hour. Scale bar, 20 μm. (B) The level of phosphorylated and total S6K in HEK293 cells pretreated with 3 uM bisindolylmaleimide I (Bis) for 1 hour prior to treatment with 100 nM PMA for 1 hour. (C) Western blot analysis of phosphorylated and total S6K in HEK cells transfected with control, PKCη, PKCδ, PKCε, or PKCtheta siRNA and then treated with 100 nM PMA 5 hours after serum starvation. Bottom panels show efficiency of siRNAs on respective types of PKCs by protein level (PKCδ, PKCε) or mRNA (PKCη, PKCtheta). (D) Confocal microscope images of immunofluorescence labeling of mTOR and LAMP2 in HEK cells transfected with control or PKCη siRNA and treated with 100 nM PMA for 1 hour. Scale bar, 20 μm and 10 μm in enlarged images. Images are representative of three fields examined from three independent experiments. Western blots are representative of at least three independent experiments.

The lack of effect of the cPKC inhibitor Gö 6976 (**Fig 3A**) on PMA-induced accumulation of mTOR in the perinuclear lysosomes raised the possibility that other members of the PKC family are regulators of mTOR translocation. As only the subtypes of classical and novel PKC are PMA sensitive, we examined the effect of bisindolylmaleimide I (Bis), an inhibitor of both cPKC and nPKC, on the mTOR localization. Bis strongly inhibited the perinuclear accumulation and colocalization of mTOR in the lysosomes and S6K phosphorylation induced by PMA (**Fig 4A and 4B**).

To define the specific nPKC isoform involved in mTORC1 translocation, an siRNA approach was employed. Transfection of siRNAs for PKCη, PKCδ, PKCε and PKC-theta significantly reduced the levels of their respective isoforms. Strikingly, while knockdown of PKCδ, PKCε, and PKC-theta had little or no effect, PKCη siRNA significantly inhibited PMA-induced S6K phosphorylation suggesting this may be the relevant isoform required for lysosomal translocation of mTOR (**Fig 4C**). Indeed, PMA could not induce the lysosomal accumulation of mTOR in the cells transfected with PKCη siRNA (**Fig 4D and 4D’**). Taken together, these data suggest that PKCη plays a major role in the accumulation of mTOR induced by PMA.

### PMA-induced translocation of mTOR to the lysosomes requires RAG, the Ragulator complex, and v-ATPase

**Fig 5:**
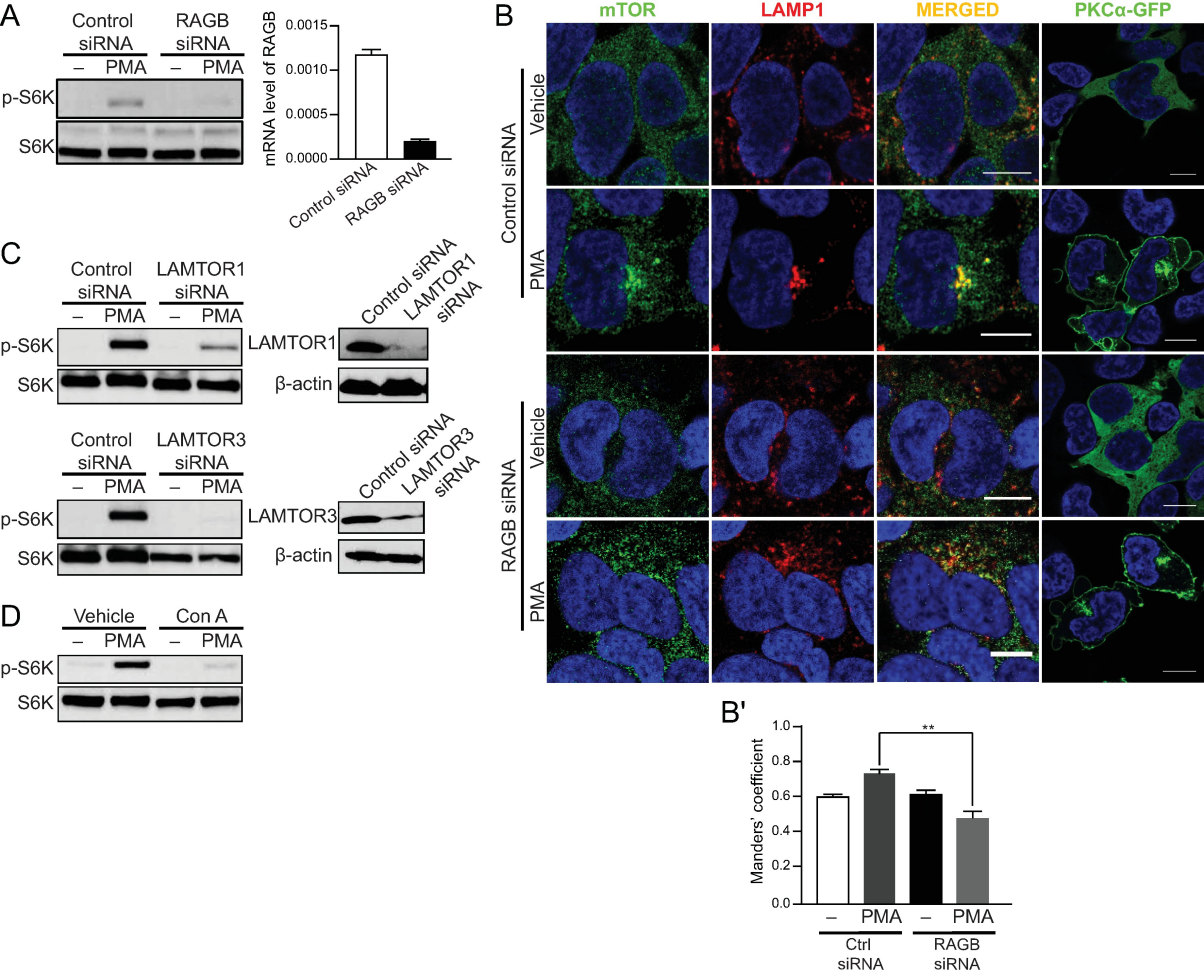
PMA-induced translocation of mTOR to the lysosomes requires RAG, Ragulator, and v-ATPase. (A) Western blot analysis of the level of phosphorylated and total S6K and (B) Confocal microscope images of immunofluorescence labeling of mTOR, LAMP1, and GFP in PKCα-GFP stably overexpressed HEK293 cells transfected with control or RAGB siRNA and treated with 100 nM PMA for 1 hour after serum starvation. Right panel shows efficiency of siRNA by *RAGB* mRNA level. Scale bar, 10 μm. (C) The level of phosphorylated and total S6K in HEK293 cells transfected with control, LAMTOR 1, or LAMTOR 3 siRNA and treated with 100 nM PMA for 1 hour after serum starvation. Right panels show efficiency of each siRNA on their targets by the protein level. (D) The level of phosphorylated and total S6K in HEK293 cells pretreated with 1 uM concanamycin A (Con A) for 1 hour followed by 100 nM PMA for 1 hour. Images are representative of three fields examined from three independent experiments. Western blots are representative of at least three independent experiments.

Research into the mechanisms underlying mTORC1 localization to the lysosomal surface has identified a number of regulatory mechanisms. While the RAG GTPases function as key proteins that recruit mTORC1 to the lysosome through interacting with RAPTOR, a number of upstream regulators of RAGs have been reported including the Ragulator complex, v-ATPase, GATOR2 complex and Sestrins (as positive regulators) with Folliculin and GATOR1 as negative regulators [4, 6–9]. Having observed the PKCη-dependent translocation of mTORC1 to the lysosomes, it was important to explore which downstream regulators might be involved. Investigating the RAGs as the most proximal regulators, RAGB was knocked down with siRNA–as verified by qRT-PCR (**Fig 5A**) and the effects on S6K phosphorylation and mTOR translocation were determined. Consistent with the essential role for RAGs in localizing mTORC1 to the lysosome, the treatment of RAGB siRNA inhibited both PMA-induced S6K phosphorylation (**Fig 5A**) and mTOR translocation to the lysosome (**Fig 5B**) while having no effect on PMA-induced translocation of PKCα to the perinuclear region (**Fig 5B**). Moving further upstream, two components of the Ragulator complex–LAMTOR1 and LAMTOR 3—were knocked down by siRNAs. As can be seen, each siRNA reduced the levels of their respective targets. Moreover, as with RAGB knockdown, effects of PMA on S6K phosphorylation were inhibited in the absence of LAMTOR1 or LAMTOR3 (**Fig 5C**). Finally, concanamycin A–an inhibitor of the v-ATPase–was utilized and, again, PMA-induced S6K phosphorylation were inhibited (**Fig 5D**). Collectively, these results place PKCs upstream of the v-ATPase/Ragulator/RAGB in regulating mTORC1 translocation to the lysosome.

## Discussion

**Fig 6:**
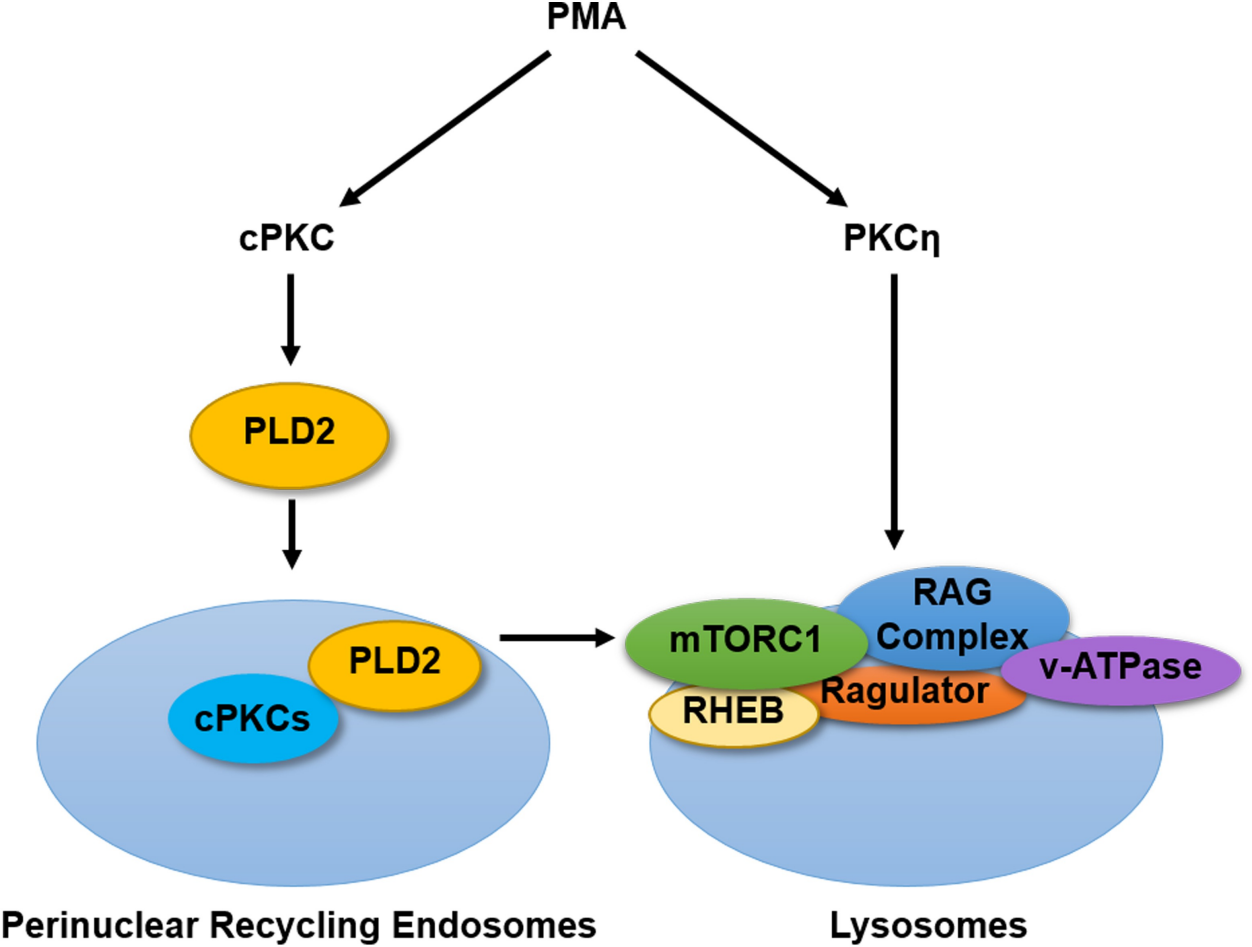
Co-ordinated activation of classical and novel PKC isoforms is required for full mTORC1 activation by PMA. A schematic summarizing the major findings of the study. PMA activation of mTORC1 requires activation of two separate pathways: a cPKC-dependent pathway that culminates in perinuclear PLD2 accumulation, and a nPKC-dependent pathway acting through PKCη that promotes mTORC1 translocation to perinuclear lysosomes.

Research to date has established the mTOR pathway as a downstream effector of PKC signaling both *in vitro* and *in vivo* yet the mechanisms by which PKCs activate mTORC1 remain unclear. In our previous study, we found that sustained activation of PKC with PMA treatment activated mTORC1 in a temporally delayed manner. Here, we have further explored the mechanisms underlying this regulation. We found that PMA-induced activation of mTORC1 requires two distinct signaling pathways through the concurrent activation of two specific classes of PKC as summarized schematically (**Fig 6**). Importantly, these results reveal a hitherto unknown role for PKCη in regulating lysosomal translocation and activity of mTORC1. Moreover, it is further confirmed that PMA activation of mTORC1 is largely independent of ERK and RSK. Collectively, these findings define novel mechanisms by which PKCs regulate mTORC1 activity and show, for the first time, that PKCs are involved in the spatial regulation of mTORC1.

The mTOR pathway is a central integrator of environmental cues and is highly regulated by multiple pathways and inputs. In recent years, the spatial regulation of mTORC1 has become appreciated beginning with the discovery that mTORC1 can cycle on and off the late endosome/lysosome surface through interactions with the RAG proteins [4]. Subsequently, numerous studies have concluded that the lysosomal localization of mTORC1 is essential for full activity in response to serum, growth factors, glucose and amino acids [36] although some reports have suggested this is cell type specific [37, 38]. Nonetheless, a primary finding of our current study identifies the nPKC isoform PKCη as an essential regulator for mTORC1 translocation to the lysosomal surface, confirmed by the use of both pharmacological inhibitors and siRNA. Importantly, our PLA results suggest that this is an increased translocation of mTORC1 to the lysosomes rather than a reflection of the clustering of lysosomes in the perinuclear region. The functional importance of this isoform in mTORC1 signaling was confirmed with siRNA against PKCη, but not PKCδ or PKCε, strongly inhibiting S6K phosphorylation.

Considerable research has defined multiple upstream pathways that converge on the Rag proteins to regulate lysosomal mTOR localization including the Ragulator complex, v-ATPase, folliculin, and the GATOR complexes amongst others [6, 8, 9, 38, 39]. Mechanistically, our results place PKCη in the v-ATPase/Ragulator pathway as PMA-induced translocation of mTORC1 to the lysosomes was inhibited by knockdown of RAGB, a component of the Ragulator complexes and the v-ATPase inhibitor, concanamycin A. Consistent with this hypothesis, previous research has shown that PMA is able to increase v-ATPase activity [40, 41] although it should be noted that, to date, no specific PKC isoforms have been implicated in this process. To expand our findings, it would be determined that PKCη is implicated in the activation of mTORC1 by amino acids and in the general regulatory upstream of mTORC1 axis in the future study.

In addition to the finding of the unappreciated effect of PMA on localization of mTORC1, we further observed that lysosomal translocation of mTORC1 alone is insufficient for full mTORC1 activity. Instead, PMA activation of mTORC1 required the co-ordinated activation of two separate pathways–the nPKC-dependent arm that induced lysosomal mTOR translocation and perinuclear accumulation of the lysosomes discussed above and a cPKC-dependent arm that results in accumulation of PKC and PLD2 in a perinuclear subset of recycling endosomes. Notably, this agrees with a number of reports suggesting that glucose, amino acids and growth factors all influence mTORC1 activity through multiple pathways [42, 43]. Importantly, these pathways were clearly mechanistically distinct as perinuclear accumulation of cPKC and PLD2 was unaffected by loss of PKCη or RAGB, while mTORC1 translocation to the lysosome was not prevented by inhibitors of classical PKC, PLD2 or endocytosis. With the growing number of stimuli reported to modulate mTORC1 activity, it would be of interest to determine their dependence on these pathways–particularly if they act independently of the classical upstream regulators of mTORC1 –as this may have therapeutic relevance. Indeed, in our previous study, we observed that increased expression of PKCβII in lung cancer cells directly engaged the PLD2 pathway to hyperactive mTORC1 [28].

A number of previous studies have defined PLD and its downstream product, phosphatidic acid as important players in the regulation of mTORC1 activity in response to serum, growth factors and amino acids. Together with our earlier study, we now extend these findings to implicate PLD activity in PMA activation of mTORC1 which strengthens the idea that PLD is a central hub of the non-lysosomal translocation arm of mTORC1 regulation. Interestingly, studies on the role of PLD in mTORC1 activation have primarily implicated the PLD1 isoform [43, 44]. In contrast, this study and our previous work [28] clearly implicate PLD2 suggesting that there may be some functional redundancy. Indeed, studies have shown that overexpression of both PLD1 and PLD2 can increase mTOR activity [44, 45]. However, there do appear to be differences in isoform localization with PLD2 in the recycling endosomes whereas PLD1 was reported to translocate on and off the lysosomes in a RAG-independent fashion [46, 47]. It is possible that this may position each isoform to promote mTORC1 activation through distinct mechanisms. Indeed, a number of mechanisms for phosphatidic acid-dependent activation of mTORC1 have been proposed including promoting assembly and stability of mTOR complexes [48], binding to mTOR and disrupting the interaction with inhibitory proteins such as DEPTOR and FKBP38 [46, 49], or modulating upstream pathways. Given this, it seems plausible to conclude that endosomal PLD2 directly influences mTORC1 in the lysosome, presumably by one of the above mechanisms although our studies also add an extra layer to this regulation as, in the context of the PMA response, PLD activity is required to bring PLD2 in close proximity to mTOR. While we have not defined how PLD2 directly influences mTORC1 activity, the current study suggests that PMA provides an additional tool with which to explore these mechanisms.

In conclusion, this study has explored the mechanisms underlying PMA activation of mTORC1. We show for the first time that there is a spatial aspect to the regulation of mTORC1 by PMA, requiring concurrent activation of cPKCs and nPKCs that serves to bring endosomal PLD2 and lysosomal mTOR in close proximity to elicit full activation. We also identify a novel upstream regulator of mTOR translocation to the lysosomes. Given that the mTORC1 pathway is often hyperactivated in many cancers and other pathologies, this opens up the targeting of PKCs, particularly the novel PKCη, as a potential avenue for therapeutic intervention.

## Abbreviations

Bis: Bisindolylmaleimide I
cPKC: Classical protein kinase C
DAG: Diacylglycerol
ERK: Extracellular related kinase
FKBP38: FK506 binding protein 38
GPCR: G-protein coupled receptor
mTOR: Mammalian target of rapamycin
mTORC1: mTOR Complex 1
nPKC: Novel protein kinase C
PLA: Proximity ligation assay
PLD: Phospholipase D
PMA: Phorbol 12,13-myristate acetate
RSK: p90 Ribosomal Kinase
S6K: p70 Ribosomal S6 Kinase
v-ATPase: vacuolar H^+^-ATPase

## References

[1] Sengupta S, Peterson TR, Sabatini DM. Regulation of the mTOR complex 1 pathway by nutrients, growth factors, and stress. Mol Cell. 2010;40(2):310–22. doi: 10.1016/j.molcel.2010.09.026 ; PubMed Central PMCID: PMCPMC2993060. 20965424 10.1016/j.molcel.2010.09.026 PMC2993060

[2] Wullschleger S, Loewith R, Hall MN. TOR signaling in growth and metabolism. Cell. 2006;124(3):471–84. doi: 10.1016/j.cell.2006.01.016 . 16469695 10.1016/j.cell.2006.01.016

[3] Sancak Y, Peterson TR, Shaul YD, Lindquist RA, Thoreen CC, Bar-Peled L, et al The Rag GTPases bind raptor and mediate amino acid signaling to mTORC1. Science. 2008;320(5882):1496–501. doi: 10.1126/science.1157535 ; PubMed Central PMCID: PMCPMC2475333. 18497260 10.1126/science.1157535 PMC2475333

[4] Sancak Y, Bar-Peled L, Zoncu R, Markhard AL, Nada S, Sabatini DM. Ragulator-Rag complex targets mTORC1 to the lysosomal surface and is necessary for its activation by amino acids. Cell. 2010;141(2):290–303. doi: 10.1016/j.cell.2010.02.024 ; PubMed Central PMCID: PMCPMC3024592. 20381137 10.1016/j.cell.2010.02.024 PMC3024592

[5] Long X, Lin Y, Ortiz-Vega S, Yonezawa K, Avruch J. Rheb binds and regulates the mTOR kinase. Curr Biol. 2005;15(8):702–13. doi: 10.1016/j.cub.2005.02.053 . 15854902 10.1016/j.cub.2005.02.053

[6] Zoncu R, Bar-Peled L, Efeyan A, Wang S, Sancak Y, Sabatini DM. mTORC1 senses lysosomal amino acids through an inside-out mechanism that requires the vacuolar H(+)-ATPase. Science. 2011;334(6056):678–83. doi: 10.1126/science.1207056 ; PubMed Central PMCID: PMCPMC3211112. 22053050 10.1126/science.1207056 PMC3211112

[7] Parmigiani A, Nourbakhsh A, Ding B, Wang W, Kim YC, Akopiants K, et al Sestrins inhibit mTORC1 kinase activation through the GATOR complex. Cell Rep. 2014;9(4):1281–91. doi: 10.1016/j.celrep.2014.10.019 ; PubMed Central PMCID: PMCPMC4303546. 25457612 10.1016/j.celrep.2014.10.019 PMC4303546

[8] Bar-Peled L, Chantranupong L, Cherniack AD, Chen WW, Ottina KA, Grabiner BC, et al A Tumor suppressor complex with GAP activity for the Rag GTPases that signal amino acid sufficiency to mTORC1. Science. 2013;340(6136):1100–6. doi: 10.1126/science.1232044 ; PubMed Central PMCID: PMCPMC3728654. 23723238 10.1126/science.1232044 PMC3728654

[9] Yoon MS, Sun Y, Arauz E, Jiang Y, Chen J. Phosphatidic acid activates mammalian target of rapamycin complex 1 (mTORC1) kinase by displacing FK506 binding protein 38 (FKBP38) and exerting an allosteric effect. J Biol Chem. 2011;286(34):29568–74. doi: 10.1074/jbc.M111.262816 ; PubMed Central PMCID: PMCPMC3190997. 21737445 10.1074/jbc.M111.262816 PMC3190997

[10] Manning BD, Cantley LC. Rheb fills a GAP between TSC and TOR. Trends Biochem Sci. 2003;28(11):573–6. doi: 10.1016/j.tibs.2003.09.003 . 14607085 10.1016/j.tibs.2003.09.003

[11] Shaw RJ. LKB1 and AMP-activated protein kinase control of mTOR signalling and growth. Acta Physiol (Oxf). 2009;196(1):65–80. doi: 10.1111/j.1748-1716.2009.01972.x ; PubMed Central PMCID: PMCPMC2760308. 19245654 10.1111/j.1748-1716.2009.01972.x PMC2760308

[12] Buller CL, Loberg RD, Fan MH, Zhu Q, Park JL, Vesely E, et al A GSK-3/TSC2/mTOR pathway regulates glucose uptake and GLUT1 glucose transporter expression. Am J Physiol Cell Physiol. 2008;295(3):C836–43. doi: 10.1152/ajpcell.00554.2007 ; PubMed Central PMCID: PMCPMC2544442. 18650261 10.1152/ajpcell.00554.2007 PMC2544442

[13] Manning BD, Tee AR, Logsdon MN, Blenis J, Cantley LC. Identification of the tuberous sclerosis complex-2 tumor suppressor gene product tuberin as a target of the phosphoinositide 3-kinase/akt pathway. Mol Cell. 2002;10(1):151–62. . 12150915 10.1016/s1097-2765(02)00568-3

[14] Menon S, Dibble CC, Talbott G, Hoxhaj G, Valvezan AJ, Takahashi H, et al Spatial control of the TSC complex integrates insulin and nutrient regulation of mTORC1 at the lysosome. Cell. 2014;156(4):771–85. doi: 10.1016/j.cell.2013.11.049 ; PubMed Central PMCID: PMCPMC4030681. 24529379 10.1016/j.cell.2013.11.049 PMC4030681

[15] Steinberg SF. Structural basis of protein kinase C isoform function. Physiol Rev. 2008;88(4):1341–78. doi: 10.1152/physrev.00034.2007 ; PubMed Central PMCID: PMCPMC2899688. 18923184 10.1152/physrev.00034.2007 PMC2899688

[16] Castagna M, Takai Y, Kaibuchi K, Sano K, Kikkawa U, Nishizuka Y. Direct activation of calcium-activated, phospholipid-dependent protein kinase by tumor-promoting phorbol esters. J Biol Chem. 1982;257(13):7847–51. . 7085651

[17] Rozengurt E. Mitogenic signaling pathways induced by G protein-coupled receptors. J Cell Physiol. 2007;213(3):589–602. doi: 10.1002/jcp.21246 . 17786953 10.1002/jcp.21246

[18] Feng X, Becker KP, Stribling SD, Peters KG, Hannun YA. Regulation of receptor-mediated protein kinase C membrane trafficking by autophosphorylation. J Biol Chem. 2000;275(22):17024–34. . 10828076 10.1074/jbc.275.22.17024

[19] Feng X, Hannun YA. An essential role for autophosphorylation in the dissociation of activated protein kinase C from the plasma membrane. J Biol Chem. 1998;273(41):26870–4. . 9756933 10.1074/jbc.273.41.26870

[20] Idkowiak-Baldys J, Baldys A, Raymond JR, Hannun YA. Sustained receptor stimulation leads to sequestration of recycling endosomes in a classical protein kinase C- and phospholipase D-dependent manner. J Biol Chem. 2009;284(33):22322–31. doi: 10.1074/jbc.M109.026765 ; PubMed Central PMCID: PMCPMC2755955. 19525236 10.1074/jbc.M109.026765 PMC2755955

[21] Idkowiak-Baldys J, Becker KP, Kitatani K, Hannun YA. Dynamic sequestration of the recycling compartment by classical protein kinase C. J Biol Chem. 2006;281(31):22321–31. doi: 10.1074/jbc.M512540200 . 16751194 10.1074/jbc.M512540200

[22] Becker KP, Hannun YA. cPKC-dependent sequestration of membrane-recycling components in a subset of recycling endosomes. J Biol Chem. 2003;278(52):52747–54. doi: 10.1074/jbc.M305228200 . 14527960 10.1074/jbc.M305228200

[23] Koivunen J, Aaltonen V, Koskela S, Lehenkari P, Laato M, Peltonen J. Protein kinase C alpha/beta inhibitor Go6976 promotes formation of cell junctions and inhibits invasion of urinary bladder carcinoma cells. Cancer Res. 2004;64(16):5693–701. doi: 10.1158/0008-5472.CAN-03-3511 . 15313909 10.1158/0008-5472.CAN-03-3511

[24] El-Osta MA, Idkowiak-Baldys J, Hannun YA. Delayed phosphorylation of classical protein kinase C (PKC) substrates requires PKC internalization and formation of the pericentrion in a phospholipase D (PLD)-dependent manner. J Biol Chem. 2011;286(22):19340–53. doi: 10.1074/jbc.M110.152330 ; PubMed Central PMCID: PMCPMC3103312. 21478146 10.1074/jbc.M110.152330 PMC3103312

[25] Moschella PC, Rao VU, McDermott PJ, Kuppuswamy D. Regulation of mTOR and S6K1 activation by the nPKC isoforms, PKCepsilon and PKCdelta, in adult cardiac muscle cells. J Mol Cell Cardiol. 2007;43(6):754–66. doi: 10.1016/j.yjmcc.2007.09.015 ; PubMed Central PMCID: PMCPMC2170873. 17976640 10.1016/j.yjmcc.2007.09.015 PMC2170873

[26] Fan QW, Cheng C, Knight ZA, Haas-Kogan D, Stokoe D, James CD, et al EGFR signals to mTOR through PKC and independently of Akt in glioma. Sci Signal. 2009;2(55):ra4 doi: 10.1126/scisignal.2000014 ; PubMed Central PMCID: PMCPMC2793677. 19176518 10.1126/scisignal.2000014 PMC2793677

[27] Aeder SE, Martin PM, Soh JW, Hussaini IM. PKC-eta mediates glioblastoma cell proliferation through the Akt and mTOR signaling pathways. Oncogene. 2004;23(56):9062–9. doi: 10.1038/sj.onc.1208093 . 15489897 10.1038/sj.onc.1208093

[28] El Osta M, Liu M, Adada M, Senkal CE, Idkowiak-Baldys J, Obeid LM, et al Sustained PKCbetaII activity confers oncogenic properties in a phospholipase D- and mTOR-dependent manner. FASEB J. 2014;28(1):495–505. doi: 10.1096/fj.13-230557 ; PubMed Central PMCID: PMCPMC3868841. 24121461 10.1096/fj.13-230557 PMC3868841

[29] Leseux L, Laurent G, Laurent C, Rigo M, Blanc A, Olive D, et al PKC zeta mTOR pathway: a new target for rituximab therapy in follicular lymphoma. Blood. 2008;111(1):285–91. doi: 10.1182/blood-2007-04-085092 . 17855629 10.1182/blood-2007-04-085092

[30] Roux PP, Ballif BA, Anjum R, Gygi SP, Blenis J. Tumor-promoting phorbol esters and activated Ras inactivate the tuberous sclerosis tumor suppressor complex via p90 ribosomal S6 kinase. Proc Natl Acad Sci U S A. 2004;101(37):13489–94. doi: 10.1073/pnas.0405659101 ; PubMed Central PMCID: PMCPMC518784. 15342917 10.1073/pnas.0405659101 PMC518784

[31] Fonseca BD, Alain T, Finestone LK, Huang BP, Rolfe M, Jiang T, et al Pharmacological and genetic evaluation of proposed roles of mitogen-activated protein kinase/extracellular signal-regulated kinase kinase (MEK), extracellular signal-regulated kinase (ERK), and p90(RSK) in the control of mTORC1 protein signaling by phorbol esters. J Biol Chem. 2011;286(31):27111–22. doi: 10.1074/jbc.M111.260794 ; PubMed Central PMCID: PMCPMC3149304. 21659537 10.1074/jbc.M111.260794 PMC3149304

[32] Kitatani K, Idkowiak-Baldys J, Hannun YA. Mechanism of inhibition of sequestration of protein kinase C alpha/betaII by ceramide. Roles of ceramide-activated protein phosphatases and phosphorylation/dephosphorylation of protein kinase C alpha/betaII on threonine 638/641. J Biol Chem. 2007;282(28):20647–56. doi: 10.1074/jbc.M609162200 . 17504762 10.1074/jbc.M609162200

[33] Yoshida S, Pacitto R, Yao Y, Inoki K, Swanson JA. Growth factor signaling to mTORC1 by amino acid-laden macropinosomes. J Cell Biol. 2015;211(1):159–72. doi: 10.1083/jcb.201504097 ; PubMed Central PMCID: PMCPMC4602043. 26438830 10.1083/jcb.201504097 PMC4602043

[34] Kikumori M, Yanagita RC, Tokuda H, Suzuki N, Nagai H, Suenaga K, et al Structure-activity studies on the spiroketal moiety of a simplified analogue of debromoaplysiatoxin with antiproliferative activity. J Med Chem. 2012;55(11):5614–26. doi: 10.1021/jm300566h . 22625994 10.1021/jm300566h

[35] Liu M, Idkowiak-Baldys J, Roddy PL, Baldys A, Raymond J, Clarke CJ, et al Sustained activation of protein kinase C induces delayed phosphorylation and regulates the fate of epidermal growth factor receptor. PLoS One. 2013;8(11):e80721 doi: 10.1371/journal.pone.0080721 ; PubMed Central PMCID: PMCPMC3823608.24244711

[36] Puertollano R. mTOR and lysosome regulation. F1000Prime Rep. 2014;6:52 doi: 10.12703/P6-52 ; PubMed Central PMCID: PMCPMC4108950. 25184042 10.12703/P6-52 PMC4108950

[37] Martin TD, Chen XW, Kaplan RE, Saltiel AR, Walker CL, Reiner DJ, et al Ral and Rheb GTPase activating proteins integrate mTOR and GTPase signaling in aging, autophagy, and tumor cell invasion. Mol Cell. 2014;53(2):209–20. doi: 10.1016/j.molcel.2013.12.004 ; PubMed Central PMCID: PMCPMC3955741. 24389102 10.1016/j.molcel.2013.12.004 PMC3955741

[38] Bar-Peled L, Schweitzer LD, Zoncu R, Sabatini DM. Ragulator is a GEF for the rag GTPases that signal amino acid levels to mTORC1. Cell. 2012;150(6):1196–208. doi: 10.1016/j.cell.2012.07.032 ; PubMed Central PMCID: PMCPMC3517996. 22980980 10.1016/j.cell.2012.07.032 PMC3517996

[39] Kim YM, Stone M, Hwang TH, Kim YG, Dunlevy JR, Griffin TJ, et al SH3BP4 is a negative regulator of amino acid-Rag GTPase-mTORC1 signaling. Mol Cell. 2012;46(6):833–46. doi: 10.1016/j.molcel.2012.04.007 ; PubMed Central PMCID: PMCPMC3389276. 22575674 10.1016/j.molcel.2012.04.007 PMC3389276

[40] Heming TA, Bidani A. Effects of myristate phorbol ester on V-ATPase activity and Na(+)-H+ exchange in alveolar macrophages. J Leukoc Biol. 1995;57(4):600–8. . 7722418 10.1002/jlb.57.4.600

[41] Nanda A, Gukovskaya A, Tseng J, Grinstein S. Activation of vacuolar-type proton pumps by protein kinase C. Role in neutrophil pH regulation. J Biol Chem. 1992;267(32):22740–6. . 1331065

[42] Dibble CC, Manning BD. Signal integration by mTORC1 coordinates nutrient input with biosynthetic output. Nat Cell Biol. 2013;15(6):555–64. doi: 10.1038/ncb2763 ; PubMed Central PMCID: PMCPMC3743096. 23728461 10.1038/ncb2763 PMC3743096

[43] Sun Y, Fang Y, Yoon MS, Zhang C, Roccio M, Zwartkruis FJ, et al Phospholipase D1 is an effector of Rheb in the mTOR pathway. Proc Natl Acad Sci U S A. 2008;105(24):8286–91. doi: 10.1073/pnas.0712268105 ; PubMed Central PMCID: PMCPMC2448829. 18550814 10.1073/pnas.0712268105 PMC2448829

[44] Fang Y, Park IH, Wu AL, Du G, Huang P, Frohman MA, et al PLD1 regulates mTOR signaling and mediates Cdc42 activation of S6K1. Curr Biol. 2003;13(23):2037–44. . 14653992 10.1016/j.cub.2003.11.021

[45] Chen Y, Rodrik V, Foster DA. Alternative phospholipase D/mTOR survival signal in human breast cancer cells. Oncogene. 2005;24(4):672–9. doi: 10.1038/sj.onc.1208099 . 15580312 10.1038/sj.onc.1208099

[46] Yoon MS, Du G, Backer JM, Frohman MA, Chen J. Class III PI-3-kinase activates phospholipase D in an amino acid-sensing mTORC1 pathway. J Cell Biol. 2011;195(3):435–47. doi: 10.1083/jcb.201107033 ; PubMed Central PMCID: PMCPMC3206351. 22024166 10.1083/jcb.201107033 PMC3206351

[47] Padron D, Tall RD, Roth MG. Phospholipase D2 is required for efficient endocytic recycling of transferrin receptors. Mol Biol Cell. 2006;17(2):598–606. doi: 10.1091/mbc.E05-05-0389 ; PubMed Central PMCID: PMCPMC1356572. 16291863 10.1091/mbc.E05-05-0389 PMC1356572

[48] Toschi A, Lee E, Xu L, Garcia A, Gadir N, Foster DA. Regulation of mTORC1 and mTORC2 complex assembly by phosphatidic acid: competition with rapamycin. Mol Cell Biol. 2009;29(6):1411–20. doi: 10.1128/MCB.00782-08 ; PubMed Central PMCID: PMCPMC2648237. 19114562 10.1128/MCB.00782-08 PMC2648237

[49] Yoon MS, Rosenberger CL, Wu C, Truong N, Sweedler JV, Chen J. Rapid mitogenic regulation of the mTORC1 inhibitor, DEPTOR, by phosphatidic acid. Mol Cell. 2015;58(3):549–56. doi: 10.1016/j.molcel.2015.03.028 ; PubMed Central PMCID: PMCPMC4427522. 25936805 10.1016/j.molcel.2015.03.028 PMC4427522

